# Editorial: Nuclear medicine advances through artificial intelligence and intelligent informatics

**DOI:** 10.3389/fnume.2024.1502419

**Published:** 2025-01-07

**Authors:** Lisa M. Duff, Kuangyu Shi, Charalampos Tsoumpas

**Affiliations:** ^1^ Cancer Research Scotland Institute, Glasgow, United Kingdom; ^2^ Universitätsklinik für Nuklearmedizin, Inselspital University Hospital Bern, University of Bern, Bern, Switzerland; ^3^ Department of Nuclear Medicine and Molecular Imaging, University Medical Center Groningen, University of Groningen, Groningen, Netherlands

**Keywords:** artificial intelligence, nuclear medicine, PET, SPECT, medical imaging

**Editorial on the Research Topic**
Nuclear medicine advances through artificial intelligence and intelligent informatics

## Introduction

The aim of this Research Topic is to compile a themed collection of current research in Artificial Intelligence (AI) and Informatics within Nuclear Medicine, presenting recent advancements, solutions to challenges, and prospects in the field. The integration of AI and other computational approaches into nuclear medicine is expected to improve both diagnostic accuracy and treatment outcomes in the coming years. However, the deployment of AI in this domain faces considerable challenges. Careful and coordinated efforts are essential to avoid hurried implementations, delays in achieving goals, or undue concerns among users. Our research topic collection addresses some of these issues and presents new, innovative uses of AI and informatics in nuclear medicine through five research articles.

A common concern regarding AI in clinical practice is the ethics and liability of errors (1). Frood et al. demonstrate the benefits of AI assisting radiological reporters as a decision-making tool, without fully replacing the human observer. This aligns with the conclusions reached by Herington et al. regarding assigned responsibility when using AI technologies (1). They explored integrating AI into radiological reporting workflows and the effects on speed, quality, and confidence. Their study, which involved PET imaging of lymphoma, included nine reporters of varying experience across three different centres. The results showed that quality was consistent with and without AI, while reporting times significantly increased. Additionally, confidence in identifying disease improved, particularly among the more experienced reporters.

As Frood et al. suggest, diagnosis can be further enhanced with AI technologies. Their approach delineated disease sites for streamlined reporting; however, another common approach is to extract quantitative information from these disease sites or across the whole body. This helps distinguish conditions from healthy controls or similarly presenting conditions, often yielding better results than qualitative analysis (2, 3). Mushari et al. address the low specificity of qualitative interpretations of PET (positron emission tomography) and CMR (cardiac magnetic resonance) when identifying cardiac sarcoidosis, compared to myocardial inflammation caused by COVID-19. They explored a combination of radiomic analysis and machine learning to determine whether these approaches could differentiate the two conditions with promising results.

Practical imaging solutions can also be provided by AI within dynamic imaging (4, 5). Kuttner et al. addressed the issue of deriving the dynamic imaging input function with arterial blood sampling in preclinical imaging and the hurdle this presents for longitudinal studies. Their method to predict the input function with deep learning shows comparable results to current standards and could provide an alternative to invasive blood sampling. Pan et al. demonstrate deep learning applications in simultaneous scanning using three radiotracers without the need for invasive arterial blood sampling as conventional methods require. Their method outperformed these methods and separated the different tracer signals better at both the voxel and ROI level.

PET also has inherent limitations in resolution and image quality compared to other modalities which can cause issues in AI applications. Data driven approaches can also help address these issues (5–7). Zhang et al. produced a locally adaptive smoothing Bayesian based model using a Laplace and Gaussian mixture prior distribution. The results, though shown only on preclinical data, produced a noise reduction compared to current methods, without compromising on resolution.

The integration of AI and informatics into nuclear medicine is undergoing a transformative time in how imaging is conducted, interpreted, and applied for improving patient outcomes. As the articles in this collection demonstrate, AI and machine learning methods offer the potential to enhance diagnostic accuracy, streamline workflows, and provide alternative solutions to a range of challenges. However, careful consideration of ethical implications, user confidence, and the accuracy of AI-assisted technologies remains essential. Progress in areas such as the use of deep learning in multi-tracer studies and data-driven improvements in image quality, highlight the importance of ongoing AI and informatic research in nuclear medicine. By addressing the current challenges and opportunities, the future of nuclear medicine can benefit, ultimately leading to more precise and personalized healthcare.
